# Total ureteral avulsion leading to early nephrectomy as a rare complication of simple lumbar discectomy; a case report

**DOI:** 10.1051/sicotj/2015031

**Published:** 2015-11-06

**Authors:** Farzad Omidi-Kashani, Seyed Mahdi Mousavi

**Affiliations:** 1 Associate Professor of Orthopedic, Fellowship of Spine Surgery, MD, Orthopedic Department, Orthopedic Research Center, Imam Reza Hospital, Mashhad University of Medical Sciences Mashhad Iran; 2 Orthopedic Resident, Orthopedic Department, Orthopedic Research Center, Imam Reza Hospital, Mashhad University of Medical Sciences Mashhad Iran

**Keywords:** Discectomy, Lumbar disc disease, Ureter, Nephrectomy, Complication

## Abstract

*Introduction*: Lumbar discectomy constitutes the most common and probably easiest spine surgery but it is not without complications. The aim of this work is to report a case with total ureteral avulsion during lumbar discectomy due to careless advancement of the pituitary rongeur.

*Methods*: A 59-year-old male presented with refractory left L5-S1 lumbar disc herniation. During the surgery, left sided total ureteral avulsion occurred. Early postoperative progressive abdominal pain was the main clue for further investigation and diagnostic work-up.

*Results*: Abdominal ultrasonography, intravenous pyelography, and abdominal contrast-enhanced computed tomography (CT) detected a left ureteral injury. Although the injury was detected early, ureteral repair or renal autotransplantation was not possible and nephrectomy was finally indicated, due to a significant ureteral loss.

*Discussion*: Careful use of discectomy instruments, avoidance of excessive advancement of pituitary rongeurs (more than 3 cm), and thorough knowledge of the relevant anatomy are critical in preventing ureteral injury.

## Introduction

Lumbar discectomy constitutes the most common and probably easiest spine surgery. If this routine surgery is not associated with appropriate caution and rules, it may lead to adverse and devastating complications. Although great vessel injury especially the aorta is usually quoted as the most serious complication of this surgery (surgeon’s nightmare), ureteral injury has rarely been reported [1, 2]. This injury is frequently a tear or laceration, but total ureteral avulsion leading to early nephrectomy has not been reported previously [3, 4]. In this report, we describe a case with total ureteral avulsion which led to early nephrectomy as an unintended complication of lumbar discectomy.

## Case report

A 59-year-old male patient was referred to the clinic with a twelve month history of chronic low back pain that radiated to the left lower extremity during the last two months. He also complained of tingling in the sole of the left foot. He was not able to walk and stand easily and his sitting position was extremely limited and painful. In physical examination, there was a left truncal shift (list) in the standing and walking position. One leg lumbar extension test was negative but left straight leg rising test was positive. Motor testing was normal while examination of deep tendon reflex revealed weakness in the left ankle reflex.

**Figure 1.**
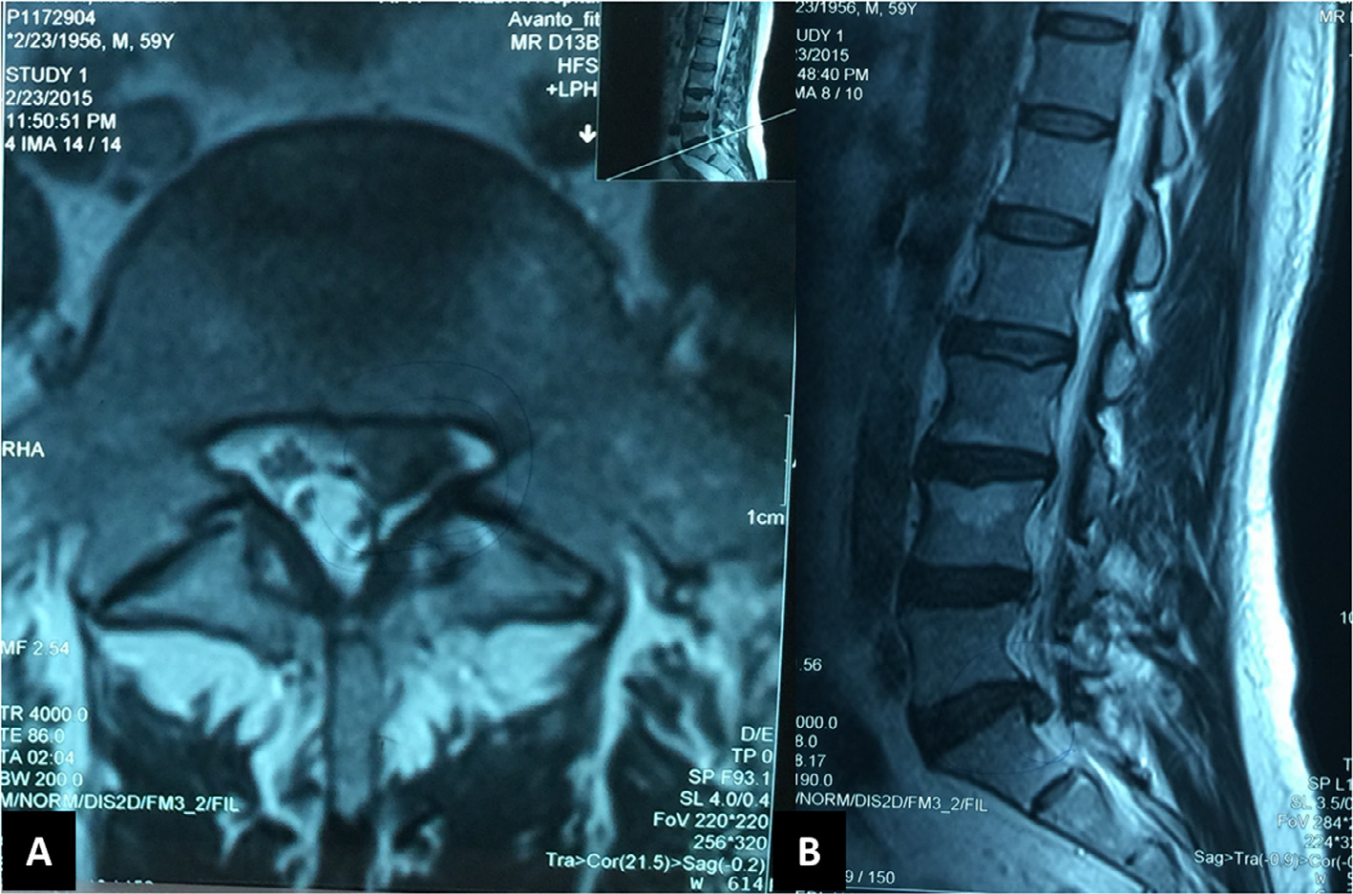
Axial (A) and sagittal (B) magnetic resonance imaging scans of the patient show degenerative disc disease in L3-L4, L4-l5, and L5-S1 levels, but left sided sequestrated disc herniation at L5-S1 was the main pathology consistent with the clinical findings.

Magnetic resonance imaging (MRI) showed degenerative disc disease at L3-L4, L4-L5, and L5-S1 levels with a sequestrated L5-S1 disc herniation at the left side (Figure 1). Plain lumbosacral radiographies did not show any underlying instability and electrophysiologic study confirmed left S1 radiculopathy. Due to the truncal shift and refractory complaints, surgical decompression was recommended to the patient. As the patient’s predominant complaint was leg pain, we planned a simple L5-S1 lumbar discectomy.

Pros and cons of the surgery were explained and the patient signed the informed consent. Surgery was performed through a minimal skin incision and sequestrated disc was easily excised. At the time we were trying to remove loose remnants of the degenerated disc, some advancement of the pituitary rongeur anteriorly was felt but no active bleeding was observed from the disc space and vital signs were completely normal and remained stable throughout the procedure. The resected tissue was sent for pathologic assessment, although it was not completely like a degenerated disc.

**Figure 2.**
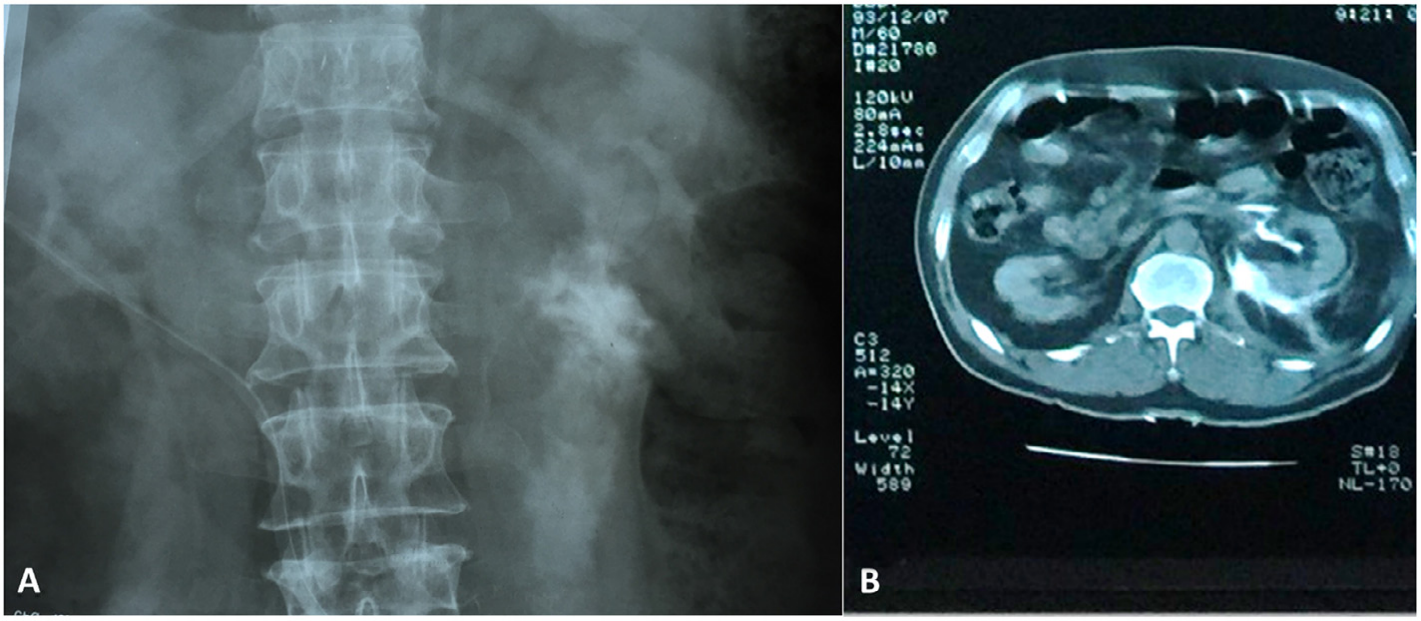
Intravenous pyelogram (A) and abdominal computed tomography (B) show fluid extravasation into the retroperitoneal space at the left side.

In the recovery room and after the patient’s anesthesia was resolved, leg pain had completely disappeared but the patient complained of some abdominal pain. Within a few hours the patient’s abdominal pain became worse and ultrasonography showed retroperitoneal fluid around the left kidney. Immediate nephrologic consultation was requested and intravenous pyelography (IVP) and abdominal contrast-enhanced computed tomography (CT) were performed (Figure 2). Imaging scans revealed left ureteral injury and the patient was immediately returned to the operating room. Surgical exploration was carried out and a 25 cm irreparable ureteral loss was detected. The ureter was abraded and avulsed from both the pelviureteric and vesicoureteric junctions. Although there are many therapeutic options including renal auto-transplantation, percutaneous nephrostomy, ileal loop reconstruction, etc., the consulted urologist decided to perform a nephrectomy based on his own experience, the intraoperative patient’s situation, and previous status of the kidney’s function. On the fifth postoperative day, the patient was discharged from the hospital with one of his kidneys lost due to an inadvertent error in surgical technique.

## Discussion

The ureters are paired muscular tubes whose peristaltic contractions propel urine from kidneys to the urinary bladder. They are about 25–30 cm in length and course down the retroperitoneal space in an *S* curve. Their proximal ends are usually adjacent to the L2 vertebra and then continue anteriorly on the psoas muscles, medial to sacroiliac joints, and then curve laterally in the pelvic. The ureters cross with the common or external iliac vessels and enter the pelvis where they are within 5 cm distance from each other.

Ureteral injuries may occur from penetrating abdominal injuries, acute deceleration injuries, and surgical procedures like hysterectomy, laparoscopic operations, or rarely during simple discectomy. Ureteral injury during posterior lumbar discectomy is usually a tear, and end-to-end anastomosis is usually possible [3]. Some authors treated ureteral injury after lumbar disc surgery with ileal ureteric replacement, ureteroureterostomy, or renal autotransplantation [1, 4]. Turunc and co-authors in 2010 reported a total ureteral avulsion due to lumbar disc surgery in a 43-year-old female. They could successfully treat the injury by end-to-end anastomosis of the ureter with an internal double J stent [2]. In this case the ureteral tube was lost during posterior lumbar discectomy and therefore, it was not amenable to repair.

Early detection of the ureteral injury plays an important prognostic role. In the absence of any indications for an emergency laparotomy, if there is a suspicion of anterior disc broaching, an abdominal angio-computed tomography (CT) for detection of nearly all the major complications associated with lumbar discectomy is strongly advocated [5].

## Conclusion

The most important stage in the treatment of ureteral injury is prevention. Careful use of discectomy instruments, avoidance of excessive advancement of pituitary rongeurs (more than 3 cm), and thorough knowledge of the relevant anatomy are critical in preventing ureteral injury. The spine surgeon should know that the integrity of the annulus fibrosus may be incomplete anteriorly and therefore, should not rely upon it for assessing the extent of rongeur advancement.

## Conflict of interest

FOK and SMM declare no conflict of interest in relation with this paper.
